# Sepsis Associated Encephalopathy

**DOI:** 10.1155/2014/762320

**Published:** 2014-09-30

**Authors:** Neera Chaudhry, Ashish Kumar Duggal

**Affiliations:** Department of Neurology, Academic Block, G.B. Pant Hospital, New Delhi 110002, India

## Abstract

Sepsis associated encephalopathy (SAE) is a common but poorly understood neurological complication of sepsis. It is characterized by diffuse brain dysfunction secondary to infection elsewhere in the body without overt CNS infection. The pathophysiology of SAE is complex and multifactorial including a number of intertwined mechanisms such as vascular damage, endothelial activation, breakdown of the blood brain barrier, altered brain signaling, brain inflammation, and apoptosis. Clinical presentation of SAE may range from mild symptoms such as malaise and concentration deficits to deep coma. The evaluation of cognitive dysfunction is made difficult by the absence of any specific investigations or biomarkers and the common use of sedation in critically ill patients. SAE thus remains diagnosis of exclusion which can only be made after ruling out other causes of altered mentation in a febrile, critically ill patient by appropriate investigations. In spite of high mortality rate, management of SAE is limited to treatment of the underlying infection and symptomatic treatment for delirium and seizures. It is important to be aware of this condition because SAE may present in early stages of sepsis, even before the diagnostic criteria for sepsis can be met. This review discusses the diagnostic approach to patients with SAE along with its epidemiology, pathophysiology, clinical presentation, and differential diagnosis.

## 1. Introduction

Sepsis is one of the most common reasons for presentation to the emergency department accounting for 6.4% of admissions [1, 2]. Sepsis and its attendant complications cause more deaths than prostate cancer, breast cancer, and HIV/AIDS combined together imposing a major financial burden on the health care system. Sepsis associated encephalopathy (SAE) is a multifactorial syndrome, characterized as diffuse cerebral dysfunction induced by the systemic response to the infection without clinical or laboratory evidence of direct brain infection or other types of encephalopathy (e.g., hepatic or renal encephalopathy). The term SAE is preferable to the loosely used term “septic encephalopathy” which to some implies a consistent, direct infection of the central nervous system. Instead the term “septic encephalopathy” might be used to define a septic state, that is, a systemic inflammatory state summoned by an infectious process of the brain or CNS. Brain dysfunction due to sepsis has been a neglected cause of delirium or altered mental status in critically ill patients primarily because there are no precise, well-established clinical or biological markers of damage to assess brain dysfunction occurring as a result of sepsis [3]. Recent studies have however reported that SAE is a relatively common cause of altered mental status in critically ill patients admitted in the ICU and its prevalence varies from 8 to 70% depending on the inclusion and exclusion criteria used [4–6]. The clinical spectrum of SAE may range from mild inattentiveness or disorientation, agitation, and hypersomnolence to more severe disturbance of consciousness as seen in coma. Although there is no direct infection or invasion of the CNS, laboratory evidence of CNS dysfunction is common in SAE. There may be evidence of abnormalities in electroencephalography (EEG) and somatosensory-evoked potentials (SSEP), increase in biomarkers such as neuron- specific enolase, S-100 *β* protein and some abnormalities on neuroimaging. None of these laboratory abnormalities are specific and SAE remains a diagnosis of exclusion and can only be diagnosed after other infectious, metabolic, and toxic causes have been ruled out by appropriate investigations. It is important to be aware of this condition because presence of encephalopathy in patients with sepsis is associated with a higher mortality rate and probable long term cognitive effects [5, 7]. The present review focuses on the clinical features, pathophysiology, differential diagnosis, and potential strategies to improve neurologic outcomes of SAE.

## 2. Sepsis: Definitions and Terminology

A prerequisite to the diagnosis of SAE is the presence of sepsis. Sepsis is defined as a clinical syndrome that complicates severe infection and is characterized by the cardinal signs of inflammation (vasodilation, leukocyte accumulation, and increased microvascular permeability) occurring in tissues that are remote from the infection. Sepsis is one of the most common reasons for admission to the medical ICUs and accounts for 37.4% of admissions [2]. American College of Chest Physicians and the Society of Critical Care Medicine (ACCP/SCCM) convened a consensus conference in 1991 to define sepsis and sepsis associated syndromes which were later modified in 2003 as shown below [8, 9]. 

*Definitions of Terms Used to Describe Sepsis and Related Syndromes*

*Systemic Inflammatory Response Syndrome.* The systemic inflammatory response to a variety of clinical insults is manifested by two or more of the following conditions [8]:

 temperature < 36°C or > 38°C,
 heart rate > 90 beats/min,
 respiratory rate > 20 breaths/min or PaCO2 < 32 mmHg,
 white blood cell (WBC) count < 4000 cells/*μ*L, > 12,000 cells/*μ*L, or > 10% immature (band forms).

*Infection.* Infection is the invasion of normally sterile tissue by organisms. 

*Bacteremia.* Bacteremia is the presence of viable bacteria in the blood. 

*Sepsis.* Documented or suspected and some of the following.

 
*General Parameters*. Fever (core temperature > 38.3°C), hypothermia (core temperature < 36°C, heart rate >  90 bpm or > 2 SD above the normal value for age, tachypnea > 30 bpm, altered mental status, significant edema or positive fluid balance (> 20 mL/kg over 24 h), and hyperglycemia (plasma glucose > 110 mg/dL or 7.7 mM/L) in the absence of diabetes.
 
*Inflammatory Parameters*. Leukocytosis (white blood cell count >  12,000/*μ*L), leukopenia (white blood cell count < 4,000/*μ*L), normal white blood cell count with > 10% immature forms, plasma C reactive protein > 2 SD above the normal value, and plasma procalcitonin > 2 SD above the normal value.
 
*Hemodynamic Parameters*. Arterial hypotension (systolic blood pressure SBP < 90 mmHg, MAP < 70 mmHg, or an SBP decrease > 40 mmHg in adults or less than two standard deviations below normal for age).
 
*Organ Dysfunction Parameters*. Arterial hypoxemia (PaO2/FiO2 < 300), acute oliguria (urine output < 0.5 mL/kg/hr for at least two hours despite adequate fluid resuscitation), creatinine increase > 0.5 mg/dL or 44.2 micromol/L, coagulation abnormalities (INR ≫ 1.5 or aPTT > 60 seconds), ileus (absent bowel sounds), thrombocytopenia (platelet count < 100,000/microL), and hyperbilirubinemia (plasma total bilirubin > 4 mg/dL.
 
*Tissue Perfusion Parameters*. Hyperlactatemia (> 1 mmol/L), decreased capillary refill, or mottling.

*Severe Sepsis (Sepsis Syndrome)*. This is sepsis complicated by organ dysfunction. So a patient with sepsis who has abnormalities of organ dysfunction parameters or tissue perfusion variables is classified as having severe sepsis. 

*Septic Shock*. It is sepsis-induced hypotension persisting despite adequate fluid resuscitation, which may be defined as infusion of 30 mL/kg of crystalloids or need vasopressors to maintain systolic blood pressure ≥ 90 mmHg or mean arterial pressure ≥ 70 mmHg. 

*Multiple-Organ Dysfunction Syndrome (MODS)*. It is progressive dysfunction of >1 organ in an acutely ill patient, such that homeostasis cannot be maintained without intervention.

Sepsis is the clinical syndrome that results from a dysregulated inflammatory response to an infection. It is defined as the presence of a probable or documented infection together with systemic manifestations of infection. In sepsis syndromes, one of the cardinal manifestations of systemic hypoperfusion is altered mental status. Patients with sepsis and encephalopathy were previously diagnosed as having septic encephalopathy. However, the use of this term is not entirely correct, because it suggests an active infection within the CNS. So the term “septic encephalopathy” is better suited to define a septic state, that is, a systemic inflammatory state summoned by an infectious process of the brain or CNS. Some authors have also used the term “delirium” instead of “encephalopathy,” but the two conditions are not synonymous since SAE is one of many causes of delirium, and delirium is not the only clinical presentation of SAE. Delirium is a fluctuating disorder of consciousness that is associated with a change in cognition or perceptual disturbance; it can be caused by any general medical condition [10]. Furthermore, symptoms of SAE can entirely bypass the stage of delirium, so that the use of 2 terms is not synonymous [11, 12].

## 3. Epidemiology

The first account of delirium associated with an infectious febrile illness was given by Dr. Jones in 1903, who proposed that cognitive dysfunction may be triggered by a febrile infectious disease that could occur during or after fever [13]. Dr. Jones was the first author to hypothesize that the same factor that caused fever could be related to brain dysfunction. Since then SAE is being increasingly recognized as a cause of cognitive dysfunction in critically ill patients. In spite of this there is a dearth of studies that have specifically addressed the incidence and prevalence of SAE [4, 14]. It is believed that SAE is the most common cause of encephalopathy in the medical-surgical ICUs, and over half of patients with sepsis have encephalopathy [15, 16]. One of the biggest problems in ascertaining the incidence and prevalence of SAE is that there is no specific test or set of diagnostic criteria to define this condition. Therefore the prevalence of SAE in various studies varies from 9% to 71% of patients with severe sepsis, depending on how it is defined [5, 16–18]. In most of these studies, the definitions of sepsis as well as criteria used to determine encephalopathy have been variable. While some earlier studies used a positive microbiological culture or self-defined clinical signs of sepsis to define sepsis most of the recent studies have used ACCP/SCCM criteria to diagnose sepsis syndrome. An equally more important limitation in studying the prevalence of SAE is the difficulty in evaluating cognition and sensorium in critically ill patients with sepsis. Various authors have used different methods to assess encephalopathy. While some authors have used clinical assessment of mental status by testing attention, memory, and orientation to define and grade encephalopathy, others have used Glasgow coma scale (GCS), slowing of the EEG, or abnormalities on cortical sensory evoked potentials to define SAE [4, 5, 16–20]. Even where clinical criteria have been used to ascertain sensorium and cognition there is a wide variability in the prevalence of SAE. Young et al. clinically assessed attention, memory, and orientation in 69 patients with fever and positive microbial culture. They found that 70% had some evidence of brain dysfunction [4]. In a larger study Sprung et al. found that an acutely altered mental status was present in 23% of patients with sepsis. This study however did not systematically use an objective testing of sensorium and the definition of sepsis was based on positive microbial cultures and 7 self-defined clinical signs [5]. In another recent study from China, the incidence of sepsis associated encephalopathy was 17.7% but this was a retrospective study and the criteria used to diagnose SAE were not clearly elucidated [14]. Clearly when more sensitive methods of testing cognition are combined with appropriate electrophysiological investigations up to 70% of patients with bacteraemia have neurological symptoms ranging from lethargy and mild inattention to coma, and almost 80% of patients have abnormalities on EEG [4, 17, 21]. These milder cases can easily be missed if scales such as GCS are used to ascertain the mental status in patients with sepsis.

The presence of SAE in a critically ill patient has prognostic implications also. In the seminal report by Sprung et al., the mortality rate of septic patients with altered mental status was 49% compared with a rate of 26% in septic patients with no neurological symptoms [5]. Eidelman et al. reported that the severity of neurological symptoms secondary to encephalopathy in patients in the ICU, as assessed by the GCS, was correlated to prognosis, with a mortality rate of up to 63% in patients who presented with GCS scores between 3 and 8 [16].

The source and the aetiology of infection are also important factors in development of SAE with biliary tract or intestinal infections being associated with greater risk of SAE followed by pulmonary infections. The most commonly implicated organisms are* Staphylococcus aureus*,* Enterococcus faecium*,* Acinetobacter* spp.,* Pseudomonas aeruginosa*, and* Stenotrophomonas maltophilia* [14]. It has been observed that patients with multiple bacteria on blood culture and with* Candida albicans* have a more severe brain dysfunction and a higher mortality rate [22].

While SAE is often described as an acute reversible syndrome, there is increasing evidence that SAE may pose substantial risks for long term cognitive impairments, including alterations in mental processing-speed, executive function, memory, attention, and visual-spatial abilities. These cognitive changes may last for several years even after recovery from sepsis and SAE and may adversely affect the functional abilities, quality of life, and the ability to return to work. This may place a tremendous burden placed on both family members and caregivers. Recent studies have concluded that 70% of sepsis survivors had neurocognitive impairments at hospital discharge, and up to 45% had neurocognitive impairments at 1 year [23].

## 4. Pathophysiology

The pathophysiology of SAE has not been established, but several likely mechanisms have been proposed [24]. SAE appears to involve direct cellular damage to the brain, mitochondrial and endothelial dysfunction, neurotransmission disturbances, and derangements of calcium homeostasis in brain tissue [25].

### 4.1. Brain Signaling during Sepsis

Brain signaling is a crucial event for the body to mount an appropriate response to invading microorganisms [26]. Detection of systemic inflammation by the brain is hampered by the blood brain barrier (BBB). Two major pathways allow neuroimmune communications: circumventricular organs (CVOs), located in the midline ventricular system, and the vagus nerve. CVOs lack a BBB thereby permitting a direct communication between brain and blood stream. Some CVOs are located in the vicinity of neuroendocrine structures (e.g., organum subfornicale, organum subcommisurale, corpus pineale, neurohypophysis, and organum vasculosum laminae terminalis), and others are located close to brainstem autonomic centers (i.e., area postrema) [27]. These CVOs express components of innate and adaptive immune systems, such as toll-like receptors, CD14, and receptors for cytokines, including interleukin-1 beta (IL-1*β*), interleukin-6 (IL-6), and tumor necrosis factor-alpha (TNF-*α*) [28–33]. The vagus nerve detects visceral inflammation through its axonal cytokines receptors, the afferent signals being relayed to the nucleus tractus solitarius in the brainstem. The activation of vagus efferent activity inhibits cytokine synthesis in damaged tissues through a cholinergic anti-inflammatory pathway (the inflammatory reflex). Once visceral or systemic inflammation is detected by the first or the second pathway, the activating signal will spread to behavioral, neuroendocrine, and neurovegetative centers. This results in increased transcription of several pro- and anti-inflammatory cytokines and chemokines in the brain. These cytokines such as TNF-*α*, IL-1*β*, transforming growth factor beta (TGF *β*), and monocyte chemoattractant protein 1 (MCP1) then modulate the expression of* α*-amino-3-hydroxy-5-methyl-4-isoxazolepropionic acid receptors (AMPARs) and N-methyl-D-aspartate receptors (NMDARs) on neurons, which can have functional consequences on cognition and behavior [34]. In addition these cytokines in particular IL-1*β* cause microglial activation which may be one of the earliest changes observed in SAE [35]. These activated glial cells acquire neurotoxic properties, notably by releasing nitric oxide, cytokines, reactive oxygen species, and glutamate thereby causing cell death within vulnerable areas of the brain. As a result this brain signaling mechanism which is actually meant to serve as a protective and anti-inflammatory mechanism becomes the culprit in the pathogenesis of SAE.

### 4.2. Microscopic Brain Injury

Direct cerebral localisation of microorganisms with formation of microabscesses has been described in human SAE [4]. However, many cases of SAE without brain microabscesses have been observed; there was no correlation between SAE and any particular microorganism making it unlikely that a direct brain action of microorganisms may play a causative role in SAE. In addition cerebral changes associated with sepsis include evidence of cerebral ischaemia and haemorrhages which can also be responsible for symptoms of SAE [36]. In a prospective postmortem study of patients with sepsis, cerebral lesions were reported which, in one patient, were compatible with multifocal necrotizing leukoencephalopathy [37].

### 4.3. Endothelial and Blood Brain Barrier Dysfunction

Adequate function of the cerebral microcirculation and BBB is important for maintenance of normal cerebral function. Under normal conditions, the BBB protects the brain from a number of insults and creates a tightly regulated microenvironment for neural cells. The integrity of the BBB is maintained by interactions between astrocytic foot processes, pericytes, and endothelial cells [38]. Experimental data indicate that, at the early phase of sepsis, endothelial nitric oxide synthase-derived nitric oxide exhibits proinflammatory characteristics and contributes to the activation and dysfunction of cerebrovascular endothelial cells [39]. Secondly lipopolysaccharides (LPS) and cytokines induced expression of adhesion molecules on brain microvessel endothelial cells also contributes to BBB dysfunction. This breakdown of the BBB facilitates the passage of neurotoxic factors such as cytokines and accounts for brain edema seen on MRI in patients with SAE [40]. BBB breakdown is more frequent around the Virchow-Robin spaces but may be diffuse throughout the white matter and posterior lobes. A higher level of CSF protein in patients with sepsis as compared to control patients without sepsis is also attributed to the breakdown of BBB [41].

### 4.4. Cerebral Microcirculation

There is an increasing body of evidence that altered cerebral microcirculation during sepsis may be responsible for the clinical manifestations of SAE. In patients with sepsis, there is disturbed cerebral autoregulation and the response of cerebral blood vessels to carbon dioxide concentration or extracellular pH is blunted [42, 43]. The loss of cerebral autoregulation makes the brain more susceptible to variations in mean arterial pressure so that critical drops in systemic blood pressure are directly transferred into the cerebral vascular bed leading to brain hypoperfusion. However, this mechanism remains controversial as some authors did not find any change in cerebral vascular autoregulation in patients with sepsis [44, 45].

### 4.5. Alterations in Neurotransmission

Several neurotransmitters appear to be related to SAE, including the cholinergic pathway and the expression of receptors for gamma-aminobutyric acid, norepinephrine, serotonin, and dopamine [46–48]. Inflammatory and metabolic changes have been proposed to be associated with sepsis and to lead to alterations in cerebral neurotransmission [49, 50]. It is proposed that cytokines such as TNF-*α* that are produced systemically during sepsis activate cerebral microglia which, in turn, release inflammatory mediators within the CNS. These inflammatory mediators cause aberrant neuronal function and, thereby, delirium and SAE [49].

Deficits in cholinergic function have been postulated to cause delirium and cognitive decline [51]. Global hypocholinergia results from several mechanisms, including impaired acetylcholine synthesis and cholinergic synaptic dysfunction (impairment of presynaptic, synaptic, or postsynaptic functions of acetylcholine). If cholinergic function is impaired as in neurodegenerative disease or because of anticholinergic medications, a more severe and long-lasting delirium could result [49]. However, in spite of this a double-blind, placebo-controlled trial showed that delirium lasts longer and mortality is increased in patients treated with the cholinesterase inhibitor rivastigmine, compared with the control group, who did not have delirium [52]. Although these findings cast a doubt on the role of cholinergic system in pathogenesis of delirium, still they do not completely rule out the possibility that disturbances in cholinergic function contribute to development of SAE. It is also possible that the imbalance between dopaminergic and cholinergic neurotransmission is responsible for delirium in critically ill patients.

The role of altered levels of serum amino acids in sepsis and its implications for possible neurotransmitter imbalance in the brain is complicated, controversial, and still unclear. Reduced levels of branched-chain amino acids (leucine, isoleucine, and valine), a group of large neutral amino acids, could be related to the increase in brain concentrations of aromatic amino acids (AAA). Encephalopathic septic patients have greater elevation of aromatic amino acids and lower concentrations of branched chain amino acids in plasma than do nonencephalopathic patients [53]. The aromatic amino acids excess is probably triggered by the extensive muscle proteolysis and reduced hepatic clearance [54]. Loss of integrity of the BBB means that there is an increased ratio of aromatic/branched chain amino acids in the CSF of patients with SAE. AAA can act as false neurotransmitters or they can induce a reduction in cerebral concentrations of norepinephrine, dopamine, and serotonin, while GABA brain levels seem to be unchanged [55, 56]. Altered concentration of neurotransmitters provokes a reduction of glucose utilization in brain regions of the serotoninergic and noradrenergic system [57].

Noradrenergic neurotransmission also might be particularly involved in SAE as dexmedetomidine, a selective agonist of* α*2-adrenoceptors expressed in the locus coeruleus, is associated with less brain dysfunction and better outcomes in septic patients compared with midazolam or lorazepam [58, 59].

### 4.6. Inflammatory Mediators and Complement System

Inflammatory cytokines and complement system are the final common pathway in the pathophysiology of brain dysfunction in SAE. Peripherally produced cytokines are responsible for endothelial damage and breakdown of the BBB. Locally produced cytokines mediate neuronal dysfunction and, ultimately, cell death. TNF-*α* appears to be one of the most significant inflammatory mediators in SAE. TNF-*α* induces neutrophil infiltration of the brain tissue, neuronal cell apoptosis, and brain edema (likely by inducing the expression of aquaporin 4) [60]. IL-6 also plays a crucial role in pathogenesis of SAE. IL-6 induces cyclooxygenase 2 in the glial cells thereby increasing the synthesis of prostaglandins and in particular of prostaglandin E2 which is responsible for the activation of hypothalamus pituitary adrenal axis, thereby causing fever and behavioral alterations [61]. The complement cascade also plays a role in the pathogenesis of SE. Excessive complement activation can cause altered expression of TLR4 and subsequent alterations in TNF-*α*, inducible nitric oxide synthetase (iNOS), and aquaporin 4 thereby causing edema, cell necrosis, or neuronal apoptosis [38, 62].

### 4.7. Oxidative Stress, Mitochondrial Dysfunction, and Apoptosis

Sepsis is associated with mitochondrial dysfunction, which can have remarkable consequences for cells and the health of the host. Early sepsis is associated with a decrease in mitochondrial ATP generation, which is likely mediated by cytokines, reactive oxygen species (ROS), and nitric oxide (NO) [63]. As mentioned previously altered brain signaling and BBB dysfunction lead to a sustained intracerebral presence and production of inflammatory molecules. Lipopolysaccharide produced by the Gram negative bacteria along with the various cytokines upregulates iNOS in astrocytes and other cells. The consequent increased production of NO and ROS has several deleterious effects on the neurons. NO and ROS are responsible for protein nitrosylation, impairment of long term potentiation, and inhibition of mitochondrial respiration, all of which cause pathological processes in nerve cells and an increase in apoptosis [64–66]. Mitochondrial dysfunction induced by NO is in part caused by decreased affinity of cytochrome c oxidase for oxygen and is responsible for both the induction of neural cell apoptosis and an insufficient energy supply to the neurons [11].

### 4.8. Calcium Homeostasis

SAE is associated with an increase in intracellular calcium levels. The alteration of calcium homeostasis impairs learning memory and cognitive function [11].

**Figure 1:**
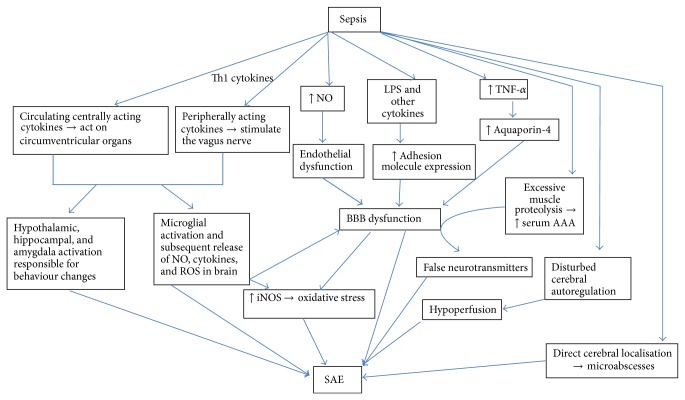
Pathophysiology of sepsis associated encephalopathy. Altered brain signalling during sepsis occurring as a result of centrally and peripherally acting cytokines results in production of various inflammatory cytokines that cause activation of various behavioral, neuroendocrine, and neurovegetative centers which can cause altered behaviour. In addition these cytokines cause microglial activation thus perpetuating the production of inflammatory cytokines and reactive oxygen species. Peripherally produced LPS, cytokines, and NO cause further damage to the BBB thereby causing a vicious cycle of brain damage. The excessive production of LPS and cytokines causes increased production of NO and other ROS thereby causing mitochondrial dysfunction and apoptosis. Altered amino acid balance because of excessive muscle proteolysis is responsible for production of false neurotransmitters which can also contribute to the pathogenesis of SAE. Disturbed cerebral autoregulation and direct cerebral localization may play a minor role. NO: nitrous oxide, ROS: reactive oxygen species, LPS: lipopolysaccharide, TNF-*α*: tumor necrosis factor-*α*, AAA: aromatic amino acids, and BBB: blood brain barrier.

To summarize the pathogenesis of SAE is complex and multifactorial (Figure 1). The brain dysfunction during sepsis can also be attributed to metabolic disturbances caused by hepatic and/or renal failure or hypoperfusion consequent to septic shock.

## 5. Clinical Features

The cardinal feature of SAE is a diffuse disturbance in cerebral function without any lateralizing signs. Two key prerequisites for making a diagnosis of SAE are presence of extracranial infection and impaired mental state. The primary clinical feature of SAE thus is a change in mental status, especially that of awareness/consciousness and cognition. SAE can intervene early in the course of a sepsis or may occur later in the course of illness as part of the multiorgan dysfunction seen in the setting of refractory septic shock [67]. Based on psychomotor activity, SAE can be differentiated into two types of presentation. The first type is characterized by agitation, confusion, disorientation, and irritability, while the second type is characterized by hypersomnolence, stupor, and coma. These two types of encephalopathic presentations are almost equally divided in a group of patients with SAE. Confusional states, lack of attention, inappropriate behaviour, confusion, disorientation, and irritability are typically seen in early form of SAE along with cardinal signs of SIRS and sepsis. When cognitive disorders are part of a late SAE, it is more common to observe more severe derangements, such as delirium and severe agitation. Impaired consciousness in the form of excessive somnolence, stupor, or coma is more often seen in the presence of multiorgan dysfunction and refractory septic shock [68]. Patients with SAE have a level of consciousness that is out of proportion to any sedative treatment they might be receiving. Another important clinical point that makes the diagnosis of sepsis associated encephalopathy difficult is that some patients might develop encephalopathy in the early phases of infection when criteria for sepsis can be met, or a confused state may develop in patients with a remote infection, such as an abdominal, walled-off abscess that has not caused bacteremia or sepsis.

In many patients the first symptoms usually appear in the early stage of sepsis often before other organ disturbances are diagnosed and include weakness, anorexia, malaise, and concentration deficits which can often be overlooked unless the clinician is aware of their significance. As sepsis progresses, mildly encephalopathic patients demonstrate a fluctuating confusional state and inappropriate behavior. They have poor attention span and may make writing errors (including spelling, writing full sentences, and orientation of writing on the page) [69]. They may also show disturbances of sleep-wake cycles or evidence of hallucinations, restlessness, or agitation, among other symptoms commonly seen in delirium. However, as has been mentioned earlier, SAE may entirely bypass the stage of delirium [11]. As sepsis becomes progressively severe and refractory, a state of multiorgan failure ensues which can further contribute to the severity of encephalopathy often culminating in coma.

Motor signs are rarely observed in septic patients. The most common motor sign is paratonic rigidity or gegenhalten, a resistance to passive movement of limbs that is velocity-dependent: the resistance felt during movements at normal rate disappears when the limb is moved slowly. Asterixis, multifocal myoclonus, seizures, and tremor are relatively infrequent as compared to other metabolic encephalopathies such as hepatic and uremic encephalopathies or dyselectrolytemia. Almost 70% of advanced cases of SAE have an associated critical illness neuropathy or myopathy or a combination of both which may be responsible for a failure to wean off these patients from mechanical ventilation [16]. The neuropathy is axonal in type and takes many months to resolve. It is later in onset and much slower to recover than the encephalopathy. Clinically, one finds decreased movement and the loss of deep tendon reflexes. There is relative preservation of cranial nerve function so that an afflicted patient will grimace at painful stimuli but only have weak limb withdrawal. The phrenic nerves are however typically involved, leading to trouble weaning from mechanical ventilation. As mentioned above cranial nerve functions are almost invariably spared, even in severe cases of SAE including those associated with critical illness neuropathy or myopathy, which distinguishes it from the locked-in syndrome and Guillain-Barré syndrome. Signs of lateralization such as gaze palsy or a hemiparesis are extremely rare and mandate prompt exclusion of more frequent causes such as a brain abscess, stroke, or tumors. Hyperventilation in SAE is often due to respiratory alkalosis in the early, delirious phase and because of metabolic acidosis in advanced-stage sepsis [70].

Neuroendocrine dysfunction (e.g., blunted hypothalamic pituitary axis responses and relative vasopressin deficiency) and autonomic failure (e.g., sudden increase or drop in blood pressure and heart rate, arrhythmia, irregular respiratory pattern, and neurogenic pulmonary oedema) are some unrecognized manifestation of brain dysfunction in sepsis [45, 71–73].

## 6. Approach to Diagnosis of SAE: Screening Tools and Laboratory Investigations

**Figure 2:**
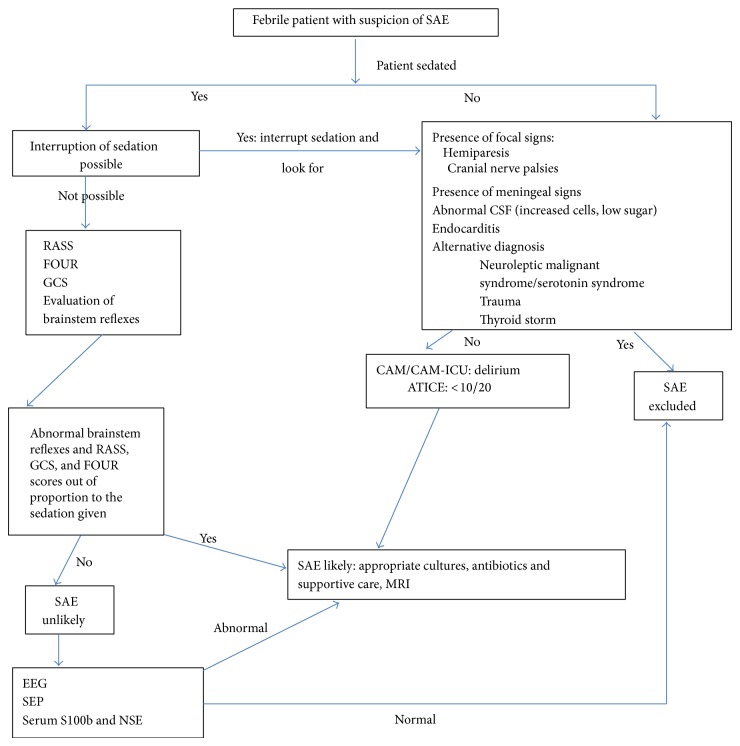
Algorithm for diagnosis of sepsis associated encephalopathy. RASS: Richmond Agitation-Sedation Scale, FOUR: Full Outline of Unresponsiveness, GCS: Glasgow coma scale, SEP: somatosensory evoked potentials, NSE: neuron specific enolase, CAM/CAM-ICU: Confusion Assessment Method (Intensive Care Unit), and ATICE: Assessment to Intensive Care Environment.

SAE is a diagnosis of exclusion and requires the exclusion of direct infection of the central nervous system, multisystem organ failure, head trauma, fat embolism, and drugs side effects. The first step in evaluation of suspected SAE would be to assess the mental status and identify features of encephalopathy which could be subtle particularly in early stages and can be confounded by the common use of sedatives in this population. The next step is to investigate and eliminate the possibility of a primary CNS pathology that may be responsible for an altered level of consciousness. Last but not least, evaluation should be aimed at identifying the source of infection and the responsible infectious agent (Figure 2).

There are several validated scores for detecting delirium and monitoring awareness in patients with SAE. In nonsedated patients mental status is straightforwardly assessed by the Confusion Assessment Method (CAM) for patients outside the ICU and Confusion Assessment Method for the Intensive Care Unit (CAM-ICU) for patients admitted to the ICU. The CAM has been validated for use in diagnosis of delirium in this setting and shows excellent sensitivity (94–100%) and positive predictive value (91–94%) [74, 75]. CAM-ICU however has only about 41–47% sensitivity when used by regular ICU nurses, although the specificity was excellent (98%) [76, 77]. CAM is most likely to miss patients with hypoactive delirium.

The Assessment to Intensive Care Environment (ATICE) score, which enables assessment of awakening, comprehension, and calmness, may indicate delirium when the score is less than 10 (maximum value being 20). The ATICE score relies heavily on assessment of eye-opening to a variety of stimuli whereas the CAM-ICU uses a broader range of responses in determining the presence of delirium or coma [78]. Unstructured delirium assessments or the Intensive Care Delirium Screening Checklist also seems to have greater sensitivity for detecting delirium in the ICU than does the CAM-ICU [79, 80]. Thus in spite of a number of screening tools, the most appropriate delirium screening tool for ICU is still controversial. Moreover all these tools assess delirium and not SAE; therefore no single tool is capable of diagnosing the full spectrum of SAE which will require a multifocal approach.

In sedated patients daily interruption of sedation may facilitate assessment of mental status [81]. However, discontinuation of sedative agents does not always result in successful awakening of the patient, and in many cases awakening is accompanied by agitation again confounding the mental examination. Moreover in these situations the persistence of coma or occurrence of agitation cannot be attributed to SAE because it can be due to the accumulation of sedatives or withdrawal from sedatives [12]. Also it may not always be feasible to interrupt sedation in critically ill patients because of fear of self-extubation and poor patient-ventilator synchronization [82]. Sedation status and its effect on mentation can be evaluated by the validated Richmond Agitation-Sedation Scale (RASS) [83]. In intubated patients FOUR (Full Outline of Unresponsiveness) scale and GCS can be used to assess the brain dysfunction. FOUR is more suitable for intubated ICU patients than is the GCS although even GCS can be useful in predicting the course of SAE and has significant prognostic value [84]. In sedated and intubated patients the loss of selected brainstem responses such as oculocephalic and cough reflex can be predictive of mortality and altered mental status and mortality. Sharshar and colleagues observed that absent oculocephalic response when adjusted for Simplified Acute Physiology Score II score, were independently associated with altered mental status after the withdrawal of sedation. Additionally an absent cough reflex was associated with a higher 28-day mortality [85].

Once brain dysfunction is identified, it is essential to do an exhaustive neurological examination assessing neck stiffness, motor responses, muscular strength and tone, plantar and deep tendon reflexes, and cranial nerves so as to rule out other causes of encephalopathy. The examination of eye position and movement, pupillary size, blinking to strong light, light response, corneal reflex, grimacing to painful stimulation, oculocephalic response, and cough reflex can be of particular importance particularly in sedated patients [12]. Examination of ocular position and movements on oculocephalic manoeuvre can be of particular help in identifying focal brainstem lesions which may be responsible for the altered consciousness in these patients. Another critical point in evaluation of patients with suspected SAE is a proper evaluation of the drugs that the patient is receiving. It should be noted that besides the obvious culprits such as benzodiazepines, opiates, anticonvulsants, and anticholinergics several other classes of drugs including antibiotics (particularly penicillins, cephalosporins, carbapenems, and quinolones), antiarrhythmics, steroids, and nonsteroidal anti-inflammatory drugs may be associated with brain dysfunction in critically ill patients [35]. A history of overdose of or withdrawal from psychoactive medications might suggest an alternative aetiology for reduced level of consciousness or delirium, and a recent history of infection requiring antibiotics can help to pinpoint a source of sepsis and thereby lead to diagnosis of SAE [86].

A thorough physical examination to investigate a source of infection (e.g., infection of a decubitus ulcer or other types of cellulitis or rash) should be supplemented with appropriate investigations such as ultrasound, echocardiography, and CT scan to look for an occult source of infection. The use of appropriate cultures (blood cultures, respiratory or sputum culture, urine culture, and cerebrospinal fluid [CSF] analysis and culture) and serologies to identify the responsible organism cannot be underemphasized. The absence of bacteraemia does not exclude SAE, and identification of a pathological organism is not always possible. Common reasons for difficulty in identifying a disease-causing organism include prior antibiotic use, which might hinder detection of specific organisms, or presence of an occult abscess [86].

A comprehensive metabolic panel of analyses including a complete blood count, measurement of expanded electrolytes (sodium, potassium, chloride, magnesium, phosphate, and calcium) and serum enzyme levels (alanine and aspartate aminotransferases, alkaline phosphatase, and *γ*-glutamyltransferase), and renal function tests (serum creatinine) should be done to look for evidence of organ dysfunction or other abnormalities that may contribute to the change in sensorium. Some of the metabolic parameters may have prognostic significance as well and it has been seen that levels of serum urea, creatinine, bilirubin, and alkaline phosphatase show a direct proportional change with the severity of the encephalopathy [22]. Finally any focal neurological abnormalities such as hemiparesis or cranial nerve abnormalities might suggest a focal neurological process, such as an abscess or a stroke and warrant urgent neuroimaging.

If the clinical examination is unrevealing, the diagnosis of SAE should rely on laboratory investigations, which may include EEG and sensory evoked potentials (SEPs). EEG is extremely sensitive for diagnosis of SAE and can show abnormalities even when the neurological examination is normal. The specificity is however poor and similar findings can be seen in other encephalopathies including hepatic and uremic encephalopathies. Young et al. studied 69 septic patients, 49 of whom had some degree of encephalopathy that was categorized as either mild or severe. They identified 5 classes of progressively worsened EEG pattern related to worsened outcome: 1: normal EEG, 2: excessive theta, 3: predominant delta, 4: triphasic waves, and 5: suppression or burst suppression, in ascending order of severity [17]. Mortality was also directly related to the severity of EEG abnormality: 0% with normal EEG, 19% with theta, 36% with delta, 50% with triphasic waves, and 67% with suppression or burst suppression. In spite of the fact that a suppression or burst suppression EEG pattern implies a poor prognosis, Young and colleagues also observed complete recovery in a few patients who had this EEG pattern thus signifying that, unlike anoxic-ischemic encephalopathy, this pattern may not always indicate a grave prognosis in SAE and recovery is possible with adequate treatment [17].

Oddo et al. observed periodic epileptiform discharges as well as seizure activity on electroencephalogram recordings in 22% of SAE patients, but, in two-thirds of the patients, the electroencephalographic abnormalities did not correlate with clinical observations. They found that seizures during continuous EEG were purely electrographic (no detectable clinical correlate) in the majority (67%) of patients. The only predictor of presence of nonconvulsive seizure activity and periodic discharges was presence of sepsis and the presence of seizures or PEDs was associated with death or severe disability at hospital discharge [87]. It has been observed that EEG may reveal brain abnormalities in 50% of patients who have no clinical evidence of encephalopathy (with relatively preserved cognition) on physical examination but have laboratory evidence of bacteraemia. These changes on EEG may resolve when sepsis is treated successfully [17].

Additionally EEG may be helpful in aiding the diagnosis of SAE because it can exclude nonconvulsive status epilepticus as a cause of altered sensorium in a critically ill patient. So an EEG should always be performed systematically in all septic patients whenever there are abnormal movements, or if delirium is suspected to rule out nonconvulsive status epilepticus. It is again important to be aware of the fact akin to clinical evaluation; sedatives may also interfere with the interpretation of signal abnormalities on the EEG.

Abnormal SEPs may also be a useful electrophysiological indicator of SAE. The benefit of using SEPs is that they are not affected by continuous sedation but, on the down side, evaluation of SEPs may be too cumbersome to be used routinely in the ICU [18]. Nevertheless, measurements of short-latency and long-latency SEP provide a valuable estimation of SE severity. Zauner et al. recorded SEP in septic patients and found that there was an increase of SEPs peak latencies in subcortical pathways (34% of all cases) and in cortical pathways (84% of all cases). This impairment of subcortical and cortical pathways was associated with severity of illness, although results were not significantly different between patients with severe sepsis and those with septic shock [18].

Cerebrospinal fluid analysis in SAE shows only a mild elevation of protein concentration in some, but not all, of the patients and normal cell counts and glucose concentrations, even in patients with brain microabscesses at autopsy [22]. In spite of this, the importance of CSF analysis cannot be underscored because it is essential to rule out primary CNS infection in patients suspected of having SAE.

The most significant use of neuroimaging is to rule out ischemic or hemorrhagic brain injury or other focal lesions, in cases with focal neurological signs. Otherwise neuroimaging in patients with SAE gives variable results. Some patients have normal brain MRI scans despite having SAE; others may show a myriad of abnormalities. Most common acute abnormalities seen on MRI include multiple ischaemic strokes or white matter lesions characterized by hyperintensity on fluid-attenuated inversion recovery (FLAIR) images in the centrum semiovale (primarily at the level of Virchow-Robin spaces). Occasional patients with SAE may also show various degrees of vasogenic edema [69]. This may reflect a breakdown of blood-brain barrier or could be due to loss of cerebral autoregulation [88]. Posterior reversible encephalopathy (PRE) may also be linked to sepsis and infection. In a study of 106 patients with PRE, 23.6% had infection or sepsis/septic shock. The clinical symptoms included altered mental status, seizures, and visual disturbances. PRE was a late finding occurring up to 30 days after infection [89]. MR angiography in this study revealed vasospasm and vessel “pruning,” perhaps due to diminished cerebral blood flow. Finelli et al. showed bilateral basal ganglia, thalamic, cerebellar, brainstem, and cerebral MR abnormalities in a case of SAE [90]. Assessment of the nature and extent of brain damage may also influence patient's prognosis and treatment since severity of these CNS lesions is associated with the severity of sepsis and correlates inversely with GCS scores [31]. Optimal timing of brain MRI in septic shock remains unknown but it should definitely be done in patients with focal neurological deficit and if there is an alteration in mental status. Furthermore changes on neuroimaging may be found in long term survivors of SAE and septic shock. A neuroimaging study in critically ill patients with delirium (not specifically SAE) demonstrated that a longer duration of delirium was associated with a smaller brain volume at 3 months after hospital discharge which was in turn associated with increased cognitive impairment at 12 months [91].

Significant changes also appear in the cerebral circulation with SE, and transcranial Doppler (TCD) sonography has been used to monitor cerebral vasomotor reactivity in SAE [92]. Disturbances in vascular autoregulation can be seen, especially in the early stages of SE, although TCD sonography cannot be entirely relied on as a diagnostic tool because in some instances of SE no differences in cerebral perfusion have been detected.

Serum biomarkers of brain injury have been used to diagnose and follow SAE course in septic patients. The 2 most promising markers that have been used in SAE are the neuron specific enolase and the S-100 *β* protein. In particular higher levels of S-100 *β* protein are associated with more severe septic states and its serum levels can predict an early ICU mortality. This determination can be seen as a bedside monitoring tool of neurological derangements in SAE [93, 94]. However again like the electrophysiological and neuroimaging abnormalities abnormal levels of these biomarkers just reflect pathologic processes in the brain and not the nature of pathology [93, 95]. Presence of S-100 *β* in serum of the patients reflects glial cell injury and abnormal BBB function, whereas presence of NSE, an intraneuronal enzyme, reflects neuronal injury [86]. The clinical importance of elevated serum levels of NSE and S-100 *β* in patients with SAE is questionable because of their poor sensitivity and specificity. Certainly, the discovery and use of other markers in the future remain a challenge worthy of further research.

To summarize SAE remains a diagnosis of exclusion. In patients presenting with symptoms suggestive of SAE, a rigorous investigation for other treatable aetiologies of encephalopathy, such as systemic organ dysfunction, stroke, intracranial haemorrhage, meningoencephalitis, or other metabolic disturbance, should be undertaken to avoid overlooking the cause of encephalopathy [86].

## 7. Differential Diagnosis

The differential diagnosis of encephalopathy in a febrile patient is broad and can be particularly difficult in a critically ill patient in the ICU setting (Figure 2). Encephalopathy in critically ill patients can result from a variety of infectious as well as noninfectious causes, which can be both masked and worsened by a septic process. In critically ill patients suspected of having SAE it is necessary to address all issues including metabolic parameters that may be responsible for the altered sensorium. First and foremost it is essential to rule out a primary CNS infection thereby highlighting the role of brain imaging and lumbar puncture in patients suspected of having SAE. Infective endocarditis is a very important differential and should always be a possibility in a patient with unexplained neurologic symptoms (focal neurologic sign or encephalopathy) and bloodstream infection [96]. Of note, endocarditis has to be ruled out in the presence of cerebral microbleeds on MRI [97]. A transthoracic echo may not always be sensitive and a transesophageal echo may be required if the index of suspicion is high. Wernicke's encephalopathy must always be kept in mind particularly in elderly patients and those with underlying chronic debilitating diseases, especially in the presence of ophthalmoplegia or ataxia. The significance of sedative overdose and withdrawal has been mentioned. Numerous iatrogenic and/or environmental factors also may aggravate brain dysfunction, such as use of physical restraints, excessive noise, or underexposure to light in the ICU.

## 8. Treatment Strategies

The mainstay of management of SAE relies on early detection of delirium—which may be the first manifestation of sepsis—determination of the underlying infection and organism, accurate and prompt treatment of the infection, and providing supportive care. Intravenous antibiotic therapy remains the mainstay of pharmacological treatment and should be initiated immediately after obtaining appropriate cultures, since early initiation of antibiotic therapy is associated with lower mortality [98]. The choice of antibiotics is again a controversial and complex issue and one should consider the patient's history (e.g., recent antibiotics received, comorbidities such as immunosuppression, clinical context (e.g., community or hospital acquired)), Gram stain data, and local resistance patterns while deciding the appropriate regimen [99–101]. Although few guidelines exist for the initial selection of empiric antibiotics in severe sepsis or septic shock, an appropriate empirical regimen should include broad-spectrum antibiotic coverage directed against both Gram-positive (vancomycin) and Gram-negative bacteria (piperacillin-tazobactam or imipenem-cilastatin or cefepime). If however* Pseudomonas* is also a possible pathogen, an additional antipseudomonal agent such as fluoroquinolone, aminoglycoside, or monobactam should be added to the regimen. Maximal recommended doses of antimicrobial drugs should be given intravenously, with adjustment for impaired renal function when necessary. Empirical antifungal therapy with an echinocandin (for caspofungin: a 70 mg loading dose and then 50 mg daily) or a lipid formulation of amphotericin B should be added if the patient is hypotensive, has been receiving broad-spectrum antibacterial drugs, is neutropenic, or has had a long term central venous catheter [102]. When culture results become available, the regimen can often be simplified, as a single antimicrobial agent is usually adequate for the treatment of a known pathogen since several clinical trials and two meta-analyses have failed to demonstrate superior overall efficacy of combination therapy compared to monotherapy with a third generation cephalosporin or a carbapenem [103–105]. Antimicrobial therapy should be given for a minimum of 7 to 10 days, although longer courses may be appropriate in patients who have a slow clinical response, an undrainable focus of infection, or immunologic deficiencies [106].

Treatment of encephalopathy associated with sepsis is primarily symptomatic. Nonpharmacologic approaches to prevent delirium are paramount in the management of severe sepsis and septic shock. Various strategies that have been used successfully include reinforcement of normal circadian sleep cycles; early mobilization; encouragement of oral fluids to prevent dehydration; frequent cues to emphasize date, location, and reason for hospitalization; and attention to visual and hearing impairments [107]. In the treatment of delirium, it is essential to identify and discontinue any medications with anticholinergic, histaminergic, and other psychotropic properties. It is also important to identify other modifiable risk factors such as bladder catheters and physical restraints and remove them when not medically necessary. It may be preferable to use inflatable mittens rather than conventional restraints when medically feasible, as this may allow patients to spend time out of bed and upright in a chair. Pharmacological management of delirium in patients with SAE requires judicious use of sedative and neuroleptics drugs. Low-dose neuroleptics may be used for patients to reinforce sleep cycles at night, though they should be used sparingly during the day unless psychomotor agitation is prominent and the patient poses a safety risk to himself and the staff. In general, lorazepam, a highly potent benzodiazepine, should be avoided. Dexmedetomidine, an alpha-2 agonist, appears to be better than lorazepam in patients with sepsis as patients treated with dexmedetomidine had more encephalopathy-free days, shorter time on the ventilator, and lower mortality compared to those treated with lorazepam [58]. So dexmedetomidine is probably preferred over benzodiazepines when sedation is necessary for patients with SAE. The incidence of seizures in SAE is low (less than 10%) and their occurrence should prompt investigations for other metabolic or structural causes [108]. This means that prophylactic use of antiepileptic drugs is not justified.

In this scenario it also needs to be recognized that current medical interventions used to decrease mortality in sepsis and acute respiratory distress syndrome might be associated with neurologic morbidity. Activated protein C (drotrecogin alpha) was thought to be a promising agent in the management of sepsis, septic shock, and SAE [109]. Spapen et al. demonstrated that use of APC in patients with SAE was associated with a decrease in serum S-100 *β* levels, which was used as a surrogate marker for SAE [110]. However results from an initial study which showed beneficial effect of APC in severe sepsis could not be replicated. In fact The Prospective Recombinant Human Activated Protein C Worldwide Evaluation in Severe Sepsis and Septic Shock (PROWESS-SHOCK) found that APC did not significantly reduce mortality at 28 or 90 days, as compared with placebo, in patients with septic shock and a Cochrane database review found that use of APC was associated with an increased risk of bleeding including CNS bleed without any significant benefit in mortality [111, 112]. This has led to withdrawal of the drug Xigris from the market.

Steroids may be administered to patients with sepsis and acute respiratory distress syndrome. In patients who have vasopressor-dependent septic shock, low-dose steroid replacement may be administered in those who have documented adrenal insufficiency. Steroids have also been shown to reduce posttraumatic stress syndrome [113]. Steroid exposure has been associated with increased risk of acquired neuromuscular weakness, specifically critical illness myopathy (CIM). Furthermore administration of steroids in itself may be responsible for psychosis.

Strict glucose control was commonly employed in the ICU, as maintenance of blood sugar between 81 mg/dL and 144 mg/dL has been shown to decrease morbidity and mortality [114]. However tight glucose control in septic and critically ill patients may be associated with neurologic sequelae of hypoglycemia such as seizures and encephalopathy. Moreover recent trials such as NICE-SUGAR trial have unequivocally demonstrated that intensively treated patients (target blood glucose level of 81 to 108 mg/dL) had a higher incidence of severe hypoglycemia and significantly higher 90-day mortality as compared to conventional glucose control (target blood glucose of <180 mg/dL) [115].

Although no definitive therapy exists for SAE, a number of interventions have been tried or suggested in humans and animal models of SAE. The use of anti-C5a antibody reduces blood brain barrier damage in a CLP model in rats, and C5a or C5aR blockade apparently improves mortality in experimental studies [116, 117]. Similarly glutamate release inhibitor riluzole and antioxidant ascorbate have been found to be beneficial in animal models of SAE [118, 119]. Role of branched chain amino acids was evaluated by Freund, who obtained positive results in septic encephalopathic patients treated with branched chain amino acid enriched solution. There were normalization of plasma amino acid pattern and regression of encephalopathic signs in septic patients who were given a solution containing 35% of branched chain amino acids [120]. So there is still a need for potential future therapies that are aimed at containing the CNS abnormalities, such as microcirculatory changes, altered BBB permeability, and abnormal neurotransmission.

## 9. Prognosis and Long Term Cognitive Effects of SAE

The most well-known and established neurological manifestations of sepsis are acute in onset and believed to be largely reversible with adequate treatment. However mounting evidence in both animal models and human studies suggests that substantial long term cognitive sequelae are associated with SAE. These long term cognitive changes are varied but mainly encompass psychomotor activity, deficits on visual and functional memory, verbal fluency, and visual construction [121]. Iwashyna et al. studied the cognitive impairment of older patients with severe sepsis (1194 patients, mean age of survivors 76.9 years) and found that prevalence of moderate to severe cognitive impairment increases by 10.6 percentage points among patients who survived severe sepsis [7]. The odds of acquiring moderate/severe cognitive impairment were 3.3 times as high following an episode of sepsis. Moreover, many patients who have had sepsis show depressive signs and anxiety disorders further contributing to a poor quality of life [122]. The duration of delirium in mechanically ventilated patients is an independent predictor of cognitive impairment (as measured by neuropsychological testing) at both 3 and 12 months after hospital discharge [123]. Semmler and colleagues used a comprehensive battery of neuropsychological tests to identify specific cognitive deficits in a younger cohort of patients (mean age: 55.4 years) who had survived sepsis. They found diffuse cognitive deficits in areas of working memory, attention, task switching, verbal learning, and memory and phonetic verbal fluency. The deficit in most of the domains was to the tune of −1*·*5 SD, which is consistent with a mild cognitive impairment [124]. The VISIONS cohort magnetic resonance imaging study found that longer duration of delirium in patients with sepsis was associated with smaller brain volumes up to 3 months after discharge and that smaller brain volumes in turn were associated with long term cognitive impairment up to 12 months [91]. This long term effect of SAE on cognition may be responsible for acceleration of cognitive decline in patients with preexisting dementia and acquired dementia in younger persons without any preexisting cognitive illness. The long term cognitive impairment puts a tremendous burden on the family members and caregivers since disability may last for several years after an admitting diagnosis of SAE.

## 10. Conclusion

In conclusion, sepsis remains a frequent cause of morbidity and mortality. The reported incidence of SAE varies widely based on the population studied and the definition used for diagnosing encephalopathy. Still it is widely believed that SAE is the most common type of encephalopathy that is seen within a medical ICU. SAE includes a wide variety of neurological manifestations ranging from inattention, disorientation, and agitation to delirium, stupor, and coma. In spite of recent advances in the understanding of the pathophysiology of SAE, it remains a diagnosis of exclusion in the absence of any definitive test or biomarkers. Physicians should have a high index of suspicion for diagnosis of SAE since SAE can present at any stage of sepsis syndrome and use of sedatives in this population can confound the clinical findings. Electrophysiologically, EEG and SEP add important information and correlate with SE severity but are by no means specific and results should always be interpreted in the clinical context. Neuroimaging has a key role in the evaluation of SAE as it not only excludes focal vascular and infectious disorders but also may unmask underlying leukoencephalopathy, the severity of which has prognostic significance. SAE should not be regarded as an acute reversible state and there may be long term cognitive as well as radiological deficits in survivors of SAE. The neurological outcome depends on the severity of sepsis: mild cases likely recover completely, while survivors of severe sepsis may have long term deficits. At present therapeutic choices are limited and are mainly based on symptom control. Therefore, efforts should be made to understand the multiple clinical presentations and pathophysiology of SAE to target therapies toward the mechanistic pathways of the disorder rather than to merely control the symptoms of SAE.
